# Lipid and immunophenotypic profiles in hemodialysis patients with citrate vs. acetate dialysates

**DOI:** 10.3389/fcvm.2025.1497353

**Published:** 2025-04-10

**Authors:** Diana Rodríguez-Espinosa, Elena Cuadrado-Payán, Laura Morantes, Miquel Gomez, Francisco Maduell, José Jesús Broseta

**Affiliations:** 1 Departament de Medicina, Facultat de Medicina i Ciències de la Salut, Universitat de Barcelona (UB), Barcelona, Spain; 2 Department of Nephrology and Renal Transplantation, Hospital Clínic of Barcelona, Barcelona, Spain

**Keywords:** lipids, dyslipidemia, acetate, acetate-free, citrate, hemodialysis, dialysate

## Abstract

**Background:**

Chronic kidney disease (CKD) is a significant cardiovascular (CV) risk factor, with dialysis-dependent CKD (DD-CKD) patients facing high mortality rates. Hypercholesterolemia is another crucial CV risk factor, typically managed with lipid-lowering therapy, though its efficacy in DD-CKD remains uncertain. Evidence shows mixed results regarding the benefits of statins in these patients. Citrate-based dialysates are known to reduce inflammatory biomarkers compared to acetate-based ones, potentially impacting lipid profiles and immune responses. This study aimed to determine the effects of citrate vs. acetate dialysate on lipid profiles and immunophenotypes in DD-CKD patients.

**Methods:**

This unicentric, cross-over, prospective study included 21 hemodialysis patients (10 males, 11 females, average age 62.25 years). Each patient underwent 24 dialysis sessions (12 with each dialysate) and acted as their own control. Lipid profiles, immunological parameters, and nutritional and inflammatory markers were measured before the last session with each dialysate.

**Results:**

After twelve dialysis sessions with citrate dialysate (CD), compared to acetate dialysate (AD), there was a statistically significant decline in TG and remnant cholesterol, with a decrease in HDL and an increase in LDL. Regarding immunology, C3 complement levels were higher, while CD3+ CD8+ and CD16+ 56+ lymphocytes were lower. Finally, total lymphocytes were lower with AD than with CD. We found no difference in predialysis nutritional nor inflammatory parameters except for ESR, which was higher when subjects used CD than AD.

**Conclusion:**

There are significant differences in lipid and immunophenotypic profiles with CD in comparison to AD. Interestingly, there could be an advantageous profile given the reduced amount of remnant cholesterol and TG. However, further studies are needed to understand if the observed changes lead to beneficial hard clinical outcomes in DD-CKD patients.

## 1 Introduction

Chronic kidney disease (CKD) is a substantial cardiovascular (CV) risk factor, being considered equivalent to having suffered a previous CV event (1–4). Patients with dialysis-dependent CKD (DD-CKD) have a 5-year mortality rate of approximately 50%, according to some authors (5–7), mainly due to CV causes. The dialysis fluid (i.e., dialysate) constitutes one of the main components of hemodialysis. Its components determine the direction and final concentration of the diffusive exchange of solutes through the dialyzer, aiming at the reduction of uremic toxins, as well as the correction of electrolyte and acid-base imbalances. The composition of the dialysate is as important as the dialyzer, the treatment time and the flows of both blood and dialysis fluid used during treatment. The most used dialysate is a bicarbonate based one that uses acetate as a weak acid to avoid the precipitation of calcium and magnesium carbonate. However, given the inflammatory and hemodynamic effects of acetate, there is a growing interest in the implementation of an alternative. The most used and studied alternative to acetate is citrate, however there is scant evidence and experience on how these different dialysates affect systemic physiological processes.

Hypercholesterolemia constitutes an important CV risk factor and is also a therapeutic objective to control in the general population (8–10). In this sense, low-density lipoprotein (LDL) levels are targeted to a certain threshold according to each patient's calculated risk, given the evidence on lipid control as a preventive measure both for primary and secondary CV events in those with high risk, including CKD patients (11–13). The association with mortality and cardiovascular disease between other relevant lipid biomarkers and DD-CKD has been studied in triglycerides (TG) (14, 15), high-density (HDL) (16–18) and very low-density (VLDL) lipoprotein cholesterol (19, 20), lipoprotein (a) [Lp(a)) (21–23), remnant cholesterol (24), as well as the calculation of the TG/HDL ratio (25). Unlike other high-cardiovascular-risk populations, the efficacy obtained from lipid-lowering therapy remains uncertain in DD-CKD patients. Two clinical trials and a systematic review failed to prove significant benefits from statins in these patients (26–28); nonetheless, there is data on the subgroup of dialysis patients from a randomized controlled trial and a real-life retrospective study that show a reduction in major cardiovascular events (29, 30). The discrepancy between findings is probably given to the exclusion in clinical trials of patients with very high LDL levels, their population's heterogeneity, accompanying cardiovascular morbidities, and the higher mortality risk explained in part by chronic inflammation (31–33). Interestingly, some studies in patients with DD-CKD have reported that high cholesterol is not associated with decreased mortality but with increased survival (34, 35). These findings suggest that LDL blood levels may indicate malnutrition, inflammation, or a sarcopenic state (34).

It is known that citrate-based dialysates reduce inflammatory biomarkers compared to acetate-based ones, and some authors state that this could be due to reduced oxidative stress and interfere with the immunological inflammation process. Acetate-based dialysates have also been associated with accumulating uremic toxins, membrane biocompatibility, and inflammation (36–38). There is evidence that inflammatory and immunological factors are relevant in establishing patients' CV risk. For instance, studies of lymphocyte subpopulations in non-CKD patients indicate that patients with a CD4/CD8 ratio higher than 1.5 (39) and an increased proportion of natural killer (NK) lymphocytes (40) are at a greater risk of CV problems than their counterparts. Also, the complement system, particularly C3 and the C3/C4 ratio, has been associated with CV disease and metabolic disorders (41–43). However, there is scarce data on the effect of different dialysates on the immune system.

This study aims to examine potential differences in the basic lipid profile and immunophenotype of patients on hemodialysis (HD).

## 2 Methods

### 2.1 Study design and population

This is a unicentric, cross-over, prospective study. Patients over 18 years old undergoing post-dilution online hemodiafiltration, who have been on dialysis for at least three months, receiving treatment three times a week for at least four hours each session, and maintaining a stable clinical condition during this period, were eligible for inclusion in the study. Each subject underwent 24 dialysis sessions, 12 with each dialysate acidifier, and acted as their own controls. Blood samples were retrieved predialysis on the last session with each acidifier.

All parameters of the dialysis session (calcium, sodium, and bicarbonate prescriptions; blood and dialysate flows; and dialysis duration), along with medical treatments, were kept constant throughout the study, except for the dialysate acidifier. The details of the dialysate characteristics can be found in Table 1. This study utilized Fresenius 6,008 CAREsystem™ dialysis monitors and FX CorDiax™ 60 dialyzers (Fresenius Medical Care, Bad Homburg v.d.H., Germany).

**Table 1:** Acetate and citrate dialysate components.

Components	Fresenius ACF 3A5	Fresenius smartbag CA 211.5
Sodium (mmol/L)	140	138
Potassium (mmol/L)	2	2
Calcium (mmol/ml)	1.5	1.5
Magnesium (mmol/ml)	0.5	0.5
Chloride (mmol/ml)	106	109
Acetate (mmol/L)	4	–
Citrate (mmol/L)	–	1
Glucose (g/L)	1	1

### 2.2 Variables

#### 2.2.1 Lipid parameters

We registered the lipidic profile by assessing the plasma levels of total cholesterol, LDL, TG, HDL and VLDL lipoprotein cholesterol, and Lp(a), as well as the calculation of the TG/HDL ratio, comparing the use of citrate (CD) vs. acetate (AD) as dialysate acidifier, after twelve sessions with each one.

Total cholesterol, HDL and TG were measured using enzymatic assays while Lp(a) levels were determined using immunoassays. LDL cholesterol was calculated using the Friedewald formula: LDL = total cholesterol—(HDL + TG/5), provided TG levels were below 400 mg/dl; otherwise, LDL was measured directly. VLDL was estimated as TG/5 (44, 45). Remnant cholesterol was defined as total cholesterol minus LDL-C minus HDL-cholesterol (46). All measurements were performed using automated analyzers to ensure accuracy and consistency across samples.

#### 2.2.2 Immunological parameters

The studied leucocyte populations were neutrophils, lymphocytes, neutrophil-to-lymphocyte ratio (NLR), natural killers (NK) cells, CD3 positive, CD4 positive, CD8 positive, and CD19 positive count. Complement levels (C3 and C4 levels) and the C3/C4 ratio.

The leucocyte analysis was performed using flow cytometry using whole blood samples on the Sysmex XR analyzer (Sysmex Corporation, Kobe, Japan) with software version SW 2.02. Complement levels and the C3/C4 ratio were determined using immunoturbidimetry, a technique in which antigen-antibody complexes induce changes in sample turbidity, allowing for precise quantification of complement components.

#### 2.2.3 Other parameters

Blood levels for uric acid, glucose, folic acid, vitamin B12, magnesium, prealbumin, creatine kinase (CK), total proteins, albumin, transferrin, interleukin-6 (IL-6), high-sensitive C-reactive protein (hs-CRP), D-dimer, and erythrocyte sedimentation rate (ESR) were measured using standard laboratory techniques.

Uric acid, creatin kinase, and glucose levels were determined by enzymatic methods, with colorimetric detection of reaction products. Folic acid and vitamin B12 concentrations were quantified using chemiluminescence. Magnesium was measured using colorimetric assays, while prealbumin, transferrin, and total proteins were quantified via immunoturbidimetry. Albumin levels were determined using colorimetric binding assays. IL-6 and hs-CRP levels were quantified using high-sensitivity immunoassays. D-dimer was measured via immunoassays specific to fibrin degradation products. Finally, the ESR was determined using the Westergren method (47, 48).

### 2.3 Statistical analysis

Quantitative variables are reported with mean and standard deviation when normally distributed or median and 25th and 75th percentiles if skewed. Normal distribution was assessed with the Shapiro–Wilk test, and the comparisons were made with the Student's paired *T*-test or Wilcoxon's signed-rank test, accordingly. A two-sided *p*-value ≤0.05 was considered statistically significant.

## 3 Results

Two-hundred and fifty-two sessions were performed with CD and AD in total. Twenty-one participants, consisting of 10 (47.6%) males and 11 (52.4%) females, with an average age of 62.3 ± 13.8 years (ranging from 33.1 to 82.3 years), were included in the study. Dialysis access included arteriovenous fistula for 12 patients and tunneled catheter for 9 patients. Various underlying renal conditions were observed, including diabetic kidney disease (6 patients), autosomal dominant polycystic kidney disease (2 patients), HIV nephropathy (2 patients), hypertensive nephropathy (2 patients), chronic glomerulonephritis (1 patient), chronic pyelonephritis (1 patient), renal cell carcinoma (1 patient), other cystic diseases (1 patient), and undetermined causes (5 patients). Noteworthy, six patients had a past cardiovascular history where three had unresolved valvulopathy, two had coronary artery disease and one had symptomatic heart failure with preserved ejection fraction due to hypertensive cardiomyopathy.

### 3.1 Lipidic profile results

**Figure 1:**
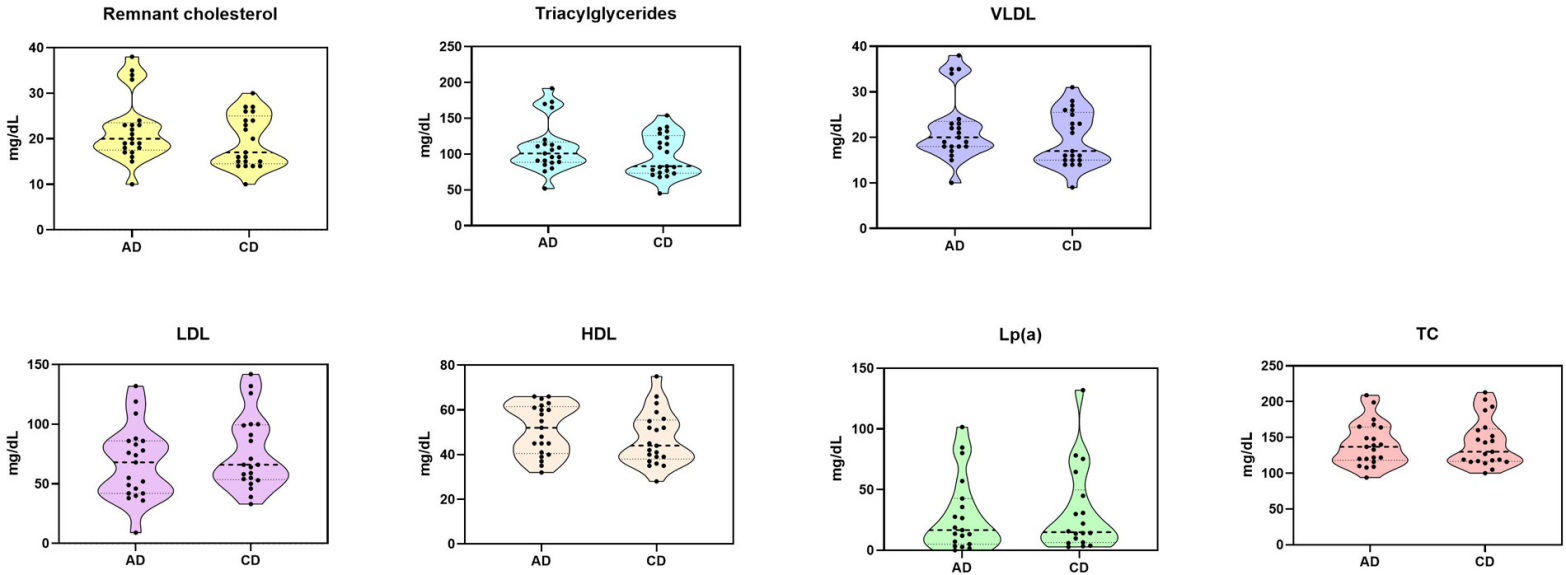
Measured lipid plasma levels after twelve dialysis sessions with each dialysate. AD, acetate dialysate; CD, citrate dialysate; HDL, high density lipoprotein; LDL, low density lipoprotein; Lp(a), lipoprotein (a); TC, total cholesterol; VLDL, very low-density lipoprotein.

There were significantly lower LDL cholesterol levels with AD compared to CD (AD: 67.05 mg/dl ± 30.53 vs. CD: 75.71 mg/dl ± 31.01, *p* = 0.042), higher remnant cholesterol [AD: 20 mg/dl (95% CI: 17.5–23.5) vs. CD: 17 mg/dl (95% CI: 17–25) *p* = 0.036], higher TG [AD: 101 mg/dl (95% CI: 88.5–117) vs. CD: 83 mg/dl (95% CI: 73.5–126) *p* = 0.046], higher HDL cholesterol (AD: 51.19 mg/dl ± 11.25 vs. CD: 47.14 mg/dl ± 11.99, *p* = 0.013). However, there were no significant differences between predialysis total cholesterol (AD: 140.33 mg/dl ± 30.56 vs. CD: 142.33 mg/dl ± 33.4, *p* = 0.624), VLDL (AD: 22.14 mg/dl ± 7.36 vs. CD: 19.67 mg/dl ± 5.97, *p* = 0.057), TG/HDL ratio (AD: 2.35 ± 1.16 and CD: 2.29 ± 1.07, *p* = 0.876), and Lp(a) values (AD: 26.21 mg/dl ± 30.45 and CD: 27.04 mg/dl ± 34.21, *p* = 0.617). See Figure 1 for further details.

### 3.2 Complement levels

We found a statistically significant difference in predialysis C3 levels between both dialysates. They were higher when patients were dialyzed with CD than with AD. However, there were no significant differences in C4 levels nor in the C3/C4 ratio (AD: 4.43 ± 0.98 vs. CD: 4.5 ± 0.92, *p* = 0.482). More details are in Table 2.

**Table 2:** Leucocyte and complement subclasses' concentrations with acetate and citrate dialysates.

Variable	Acetate	Citrate	*p*-value
Total Leucocytes × 10^3^/mm^3^, mean ± SD	7.1 ± 3.9	6.3 ± 2.2	0.03
Neutrophils × 10^3^/mm^3^, mean ± SD	4.72 ± 3.64	4.09 ± 1.75	0.412
NLR, mean ± SD	3.69 ± 3.01	3.85 ± 1.93	0.776
Lymphocytes × 10^3^/mm^3^, mean ± SD	1.37 ± 0.46	1.21 ± 0.47	0.037
CD19%, mean ± SD	121.48 ± 103.26	133.95 ± 88.31	0.423
CD3+ %, mean ± SD	72.9 ± 12.69	68.48 ± 11.86	0.005
CD8+ %, mean ± SD	33.43 ± 14.56	30.67 ± 12.85	0.05
CD4+ %, mean ± SD	517.67 ± 244.83	479.05 ± 193.74	0.272
CD4/CD8, mean ± SD	1.53 ± 1.05	1.57 ± 1.08	0.393
CD56+ CD16+ NK cells %, mean ± SD	16.95 ± 7.85	19.24 ± 8.84	0.035
C3 × 10^3^/mm^3^, mean ± SD	107.81 ± 19.71	115.14 ± 21.30	0.009
C4 × 10^3^/mm^3^, mean ± SD	25.48 ± 7.05	26.57 ± 6.78	0.097

CD, cluster of differentiation; NLR, neutrophil to lymphocyte ratio; NK, natural killer; SD, standard deviation.

### 3.3 Leucocyte count and subpopulations

There was a higher number of total leucocytes (AD: 7.1 × 10^3^/mm^3^ ± 3.9 vs. CD: 6.3 ± 2.2, *p* = 0.03) and lymphocytes (AD: 1.37 × 10^3^/mm^3^ ± 0.46 vs. CD: 1.21 ± 0.47, *p* = 0.037) with AD than with CD. We also found a higher percentage of CD3+ (AD: 72.9 ± 12.69 vs. CD: 68.48 ± 11.86, *p* = 0.0049) and CD8+ T cells (AD: 33.43 ± 14.56 vs. CD: 30.67 ± 12.85, *p* = 0.05), and higher CD56+ CD16+ NK cells (AD: 16.95 ± 7.85 vs. CD: 19.24 ± 8.84, *p* = 0.035). There were no statistically significant differences in mean CD4/CD8 ratios nor the proportion of CD4/CD8 > 1.5 with each dialysate (AD: 42.1% vs. CD: 57.9%, *p* = 0.352). There were also no differences observed in predialysis CD19+ B-cells, and CD4+ T-cells, total leucocyte count, neutrophils, NLR, monocytes, nor eosinophils between dialysates. See Table 2 for further information.

### 3.4 Inflammatory and nutritional parameters

There were no statistically significant differences in predialysis IL-6 and hs-CRP between dialysates. However, the ESR was higher when patients used CD than AD (AD: 41.19 ± 22.92 vs. CD: 53.62 ± 35.24, *p* = 0.02). Regarding nutritional parameters, we did not find any significant differences between dialysates in predialysis albumin, prealbumin, total proteins, iron, transferrin, magnesium, iron, folic acid, or vitamin B12 levels. More detailed information is available in Table 3.

**Table 3:** Inflammatory and nutritional parameters with each dialysate.

Variable	Acetate	Citrate	*p*-value
Glucose (mg/d), mean ± SD	119.15 ± 35.70	126.7 ± 59.52	0.439
Uric acid (mg/d), mean ± SD	5.36 ± 1.43	5.38 ± 1.29	0.943
Amylase (mg/d), mean ± SD	134.05 ± 85.72	125.10 ± 88.86	0.457
CK (mg/d), mean ± SD	82.24 ± 95.51	135.67 ± 204.27	0.129
Total Protein (mg/d), mean ± SD	6.79 ± 0.75	6.77 ± 0.80	0.805
Albumin (mg/d), mean ± SD	4.01 ± 0.40	3.92 ± 0.46	0.119
TSAT (mg/d), mean ± SD	24.90 ± 9.47	29.14 ± 17.19	0.132
Transferrin (mg/d), mean ± SD	177.29 ± 29.93	169.62 ± 32.03	0.066
Magnesium (mg/d), mean ± SD	2.11 ± 0.19	2.05 ± 0.26	0.192
Haptoglobin (mg/d), mean ± SD	154.67 ± 67.57	153.64 ± 75.90	0.912
Vitamin B12 (mg/d), mean ± SD	745 ± 478.39	753.62 ± 435.85	0.865
Folic acid (mg/d), mean ± SD	16.26 ± 8.23	15.90 ± 7.26	0.844
ESR (mg/d), mean ± SD	41.19 ± 22.92	53.62 ± 35.24	0.03
D-dimer (mg/d), mean ± SD	1,497.5 ± 1,614.27	1,490.56 ± 1,344.63	0.978
hs-CRP (mg/d), mean ± SD	19.58 ± 59.26	16.46 ± 29.75	0.832
Ferritin (mg/d), mean ± SD	334.67 ± 231.94	398.71 ± 246.55	0.108
Prealbumin (mg/d), mean ± SD	26.21 ± 7.59	24.89 ± 8.089	0.096

CK, creatin kinase; ESR, erythrocyte sedimentation rate; hsCRP, high-sensitivity C-reactive protein; SD, standard deviation; TSAT, transferrin saturation.

## 4 Discussion

After twelve dialysis sessions with CD, compared to AD, there was a statistically significant decline in TG, remnant cholesterol and HDL, with an increase in LDL, and a tendency towards lower VLDL. Regarding immunology, C3 complement levels were higher, while CD3+ CD8+ and CD16+ 56+ lymphocytes were lower. Finally, the total lymphocyte-count was lower with AD than with CD. We found no difference in predialysis nutritional nor inflammatory parameters except for ESR, which was higher when subjects used CD than AD.

Few studies have measured the effect of the dialysates' weak acidifier in the lipidic profile, and when done -always as secondary variables only an increase in LDL has been described with the use of CD (49). Previous studies from the 1980s did not find clinically significant changes in the lipid profile when bicarbonate solutions with reduced concentrations of acetate became commonplace over only acetate solutions (50).

Real-life findings in HD patients seem to associate lipid values with the opposite of what is expected to be beneficial in the general population. For instance, high TG levels (i.e., >193 mg/dl) correlate with a lower mortality risk (15), while high HDL levels (i.e., >60 mg/dl) correlate with an increased mortality risk (18). In our cohort, the median TG was 101 mg/dl and the highest value registered was 192 mg/dl, not reaching the apparently beneficial value previously published (15), though, it is worth noting that TG levels were significantly higher with AD than with CD. Also, unlike us, a recent study by de Sequera, et al., showed no differences in TG between AD and CD (51). With respect to remnant cholesterol, we found lower levels with CD than with AD. The median levels when patients were on AD was 20 mg/dl, whereas it was 17 mg/dl when they used CD. Levels above 39 mg/dl which have been associated with a two-fold cardiovascular mortality risk recently in a large Danish cohort (52, 53) and above 15.4 mg/dl in patients on peritoneal dialysis (24). Remnant cholesterol provides an improved correlation due to a more linear association in comparison to the U-shaped one provided by LDL, where both lower and higher blood levels are related to an increased mortality risk (54). Regarding HDL, in our cohort we found lower levels with CD than with AD, with 38% of patients having values over 60 mg/dl when using AD while only 14% crossed this threshold while on CD. With regards to LDL, even though its blood levels were higher with CD, we do not actually know the long-term clinical impact of this finding. Previous data suggest a U-shaped association between LDL. In a study performed on peritoneal dialysis patients, LDL levels above 100 mg/dl and below 85 mg/dl were associated with all-cause and cardiovascular mortality. Mean LDL levels in that population were 98 mg/dl (55) whereas our population had a much lower mean LDL of 72 mg/dl. However, as stated earlier, no clear association has been established with mortality in HD patients. As to VLDL, we found lower blood levels when patients used CD instead of AD, though not significant. There is no data on VLDL cholesterol in hemodialysis patients, however, there is data associating high VLDL with mortality in both non-dialysis dependent CKD (56) and PD patients (20), making it a potential target for treatment though more studies are required in the HD population.

Further studies must elucidate if the short-term changes induced in the lipidic profile of DD-CKD patients by the dialysate's weak acid translate into clinical implications.

Regarding immunological parameters, there is recent evidence that the use of CD for three months did not affect leucocyte nor total B or T lymphocyte count in comparison to AD in patients on HD (57). Notably, the same authors measured NLR, which has been associated with worse survival in HD patients (58), and, like us, found no differences between dialysates.

Regarding lymphocytic subpopulations, previous studies have shown that there is a U-shaped relationship between the CD4/CD8T cell- ratio and atherosclerosis progression, being associated with lower ratios in HIV patients (mean ratio of around 0.5) (59–62) and more than 1.5 in Chinese elderly population (mean ratio of around 1.33) (36). It is reported that early atherosclerotic plaques have ratios below 1, while late atherosclerotic plaques, particularly in late fibroatheroma, are around 1.5 (63). We found no differences in the proportion of low and high CD4/CD8 ratios between dialysates. However, our population's mean CD4/CD8 ratio was higher than that from the Chinese study despite their subjects being on average older than ours. This higher ratio could potentially translate into a higher CV risk, though more research is needed in dialysis patients.

We found higher total leucocytes and lymphocytes with AD than with CD, predominantly by a higher count of CD3+ and CD8+ lymphocytes, whereas NK cells were higher with CD than with AD. The interpretation of lymphocyte subpopulations needs to be clarified. There is evidence that both CD4 and CD8 decline post-hemodialysis, while their predialysis values remain unchanged compared to healthy populations (64). This is believed to be in response to poor biocompatibility with the dialyzer membrane or even with the dialysis solution, as postulated by data that has associated acetate with an increased number of activated CD3+ CD4+ CD69+ T cells than with citrate (57).

LDL receptors play a role in CD8+ T cell activation. Studies in *ldlr −/−* mice and individuals with familial hypercholesterolemia who carry a mutation in LDL receptor show decreased levels of CD8+ cytokine production and cell proliferation (60). Whether citrate alters LDL receptor function, increasing LDL levels and thus decreasing the number of CD8+ T remains unconfirmed but could partly explain the observed results compared to acetate.

Our cohort found that patients had higher circulating NK cells when exposed to CD than to AD. Activated natural killer (NK) cells utilize pyruvate, which is metabolized through the citrate-malate shuttle—a process that occurs across the mitochondrial membrane. This connection suggests that fluctuations in citrate levels could influence the metabolic reprogramming of NK cells, impacting their activation, cytotoxicity, and overall function. Citrate has been associated with promoting oxidative phosphorylation, a process essential for meeting the energy demands of NK cells during target cell elimination. This enhancement in citrate levels could potentially improve the ability of NK cells to carry out their cytotoxic functions effectively (65). NK cells constitute another subset of lymphocytes, which may also be necessary in vascular disease (66). These cells have been isolated from atherosclerotic plaques, particularly expanding the necrotic cores (37). Regarding circulating levels, a higher percentage of NK cells has been reported in patients with severe atherosclerosis awaiting revascularization (67), elderly patients with coronary disease (68), and has been associated with an increased number of CV and neurological complications after an endarterectomy (69).

Additionally, we found higher TG levels and decreased C3 levels when patients used AD than with CD. These findings may be related, given that data from experimental studies have shown that C3 stimulates glucose uptake and inhibits hormone-sensitive lipase in several cell types (40). C3 promotes lipophagy, which stimulates VLDL secretion in hepatocytes and balances TG levels in the liver (70). C3-deficient mice present with glucose intolerance delayed TG clearance and decreased TG storage (40, 71, 72). Therefore, C3 has been associated with metabolic disorders and is now recognized as a cardiometabolic risk factor (38). We also measured the C3/C4 ratio given recent data associating increased serum C3/C4 ratio as a novel marker for recurrent cardiovascular events in acute coronary syndrome (39). However, we found no differences in this parameter in our cohort.

Multiple sources state lower inflammatory parameters with CD than with AD (41–45, 73), but there are some discrepancies in the current literature (56, 74). We found no differences in IL-6 or hs-CRP, though we did find a statistically significant difference in ESR in favor of AD. It is worth noting that most of the previous evidence in this regard comes from patients on HD rather than HDF.

This study has several limitations that should be considered when interpreting the results. The single-center design, small sample size, and short follow-up limit the generalizability and long-term applicability of our findings. Although the crossover design minimizes interindividual variations, the influence of uncontrolled confounding factors, such as changes in diet, prior lipid lowering therapy, or physical activity, during the study, cannot be entirely excluded. Additionally, uncontrolled confounding factors and the absence of a non-intervention control group may have influenced the results. Larger multicenter studies are needed to validate these observations.

Based on our findings, we can conclude that using citrate dialysate in patients with DD-CKD results in significant changes to lipid profiles and immune parameters when compared to acetate dialysate. Specifically, citrate dialysate decreases TG and remnant cholesterol, lowers HDL levels and alters various lymphocyte subpopulations. These observations suggest that citrate dialysate may have distinct metabolic and immunological effects that could influence cardiovascular risk and inflammation in DD-CKD patients.

However, it remains unclear whether these changes lead to a favorable profile. While citrate dialysate reduces remnant cholesterol and has been associated with increased mortality independently of LDL-C and HDL-C levels (53), previous studies comparing mortality rates between citrate and acetate dialysate in HD patients have either shown no differences or suggested potential benefits for specific subpopulations using citrate (75). Additionally, there seems to be conflicting evidence on the role of citrate in preventing vascular calcification and magnesium supplementation and thus reducing CV morbidity and mortality (75–77). It still needs to be determined how our findings relate to those of other study groups and how they might explain certain clinical results.

To expand on our findings, future research should focus on long-term cardiovascular outcomes and immune responses. Longitudinal, multicenter trials with larger cohorts are needed to determine whether the short-term metabolic and immunological changes observed with citrate in this study result in clinically significant outcomes, such as cardiovascular event rates and mortality. Additionally, mechanistic experiments that explore the interactions among lipid metabolism, immune cell populations, and dialysate composition would provide deeper insights. Further studies that include lipidomics and immunophenotyping could help clarify the complex pathways involved, potentially guiding personalized dialysis strategies aimed at reducing cardiovascular risk and improving patient outcomes.

## Data Availability

The datasets presented in this study can be found here: https://github.com/Broseta/Citrate-dialysate.git. Further enquiries can be directed to the corresponding author.
