# Correction: lipid and immunophenotypic profiles in hemodialysis patients with citrate vs. acetate dialysates

**DOI:** 10.3389/fcvm.2025.1744975

**Published:** 2025-11-26

**Authors:** Diana Rodríguez-Espinosa, Elena Cuadrado-Payán, Laura Morantes, Miquel Gomez, Francisco Maduell, José Jesús Broseta

**Affiliations:** 1 Departament de Medicina, Facultat de Medicina i Ciències de la Salut, Universitat de Barcelona (UB), Barcelona, Spain; 2 Department of Nephrology and Renal Transplantation, Hospital Clínic of Barcelona, Barcelona, Spain

**Keywords:** lipids, dyslipidemia, acetate, acetate-free, citrate, hemodialysis, dialysate

Affiliation "Departament de Medicina, Facultat de Medicina i Ciències de la Salut, Universitat de Barcelona (UB), Barcelona, Spain" was omitted for the authors Diana Rodríguez-Espinosa, Francisco Maduell and José Jesús Broseta. This affiliation has now been added for the authors Diana Rodríguez-Espinosa, Francisco Maduell and José Jesús Broseta.

The original version of this article has been updated.

